# Regional differences in clinical phenotype of axial spondyloarthritis: results from the International Map of Axial Spondyloarthritis (IMAS)

**DOI:** 10.1093/rheumatology/kead665

**Published:** 2023-12-21

**Authors:** Denis Poddubnyy, Fernando Sommerfleck, Victoria Navarro-Compán, Christine Bundy, Souzi Makri, Shashank Akerkar, Lillann Wermskog, Elie Karam, José Correa-Fernández, Asif Siddiqui, Marco Garrido-Cumbrera

**Affiliations:** Department of Gastroenterology, Infectious Diseases and Rheumatology, Charité - Universitätsmedizin Berlin, Berlin, Germany; Rheumatology Department, German Rheumatism Research Centre, Berlin, Germany; Rheumatology Department, Sanatorio Julio Mendez, Buenos Aires, Argentina; Rheumatology Department, IdiPaz, Hospital Universitario La Paz, Madrid, Spain; Rheumatology Department, Cardiff University, Cardiff, UK; Patient Advocacy, Cyprus League for People with Rheumatism (CYLPER), Nicosia, Cyprus; Rheumatology Department, Mumbai Arthritis Clinic, Mumbai, India; Patient Advocacy, Spondyloarthritis Association of Norway (SPAFO), Oslo, Norway; Patient Advocacy, Axial Spondyloarthritis International Federation (ASIF), London, UK; Patient Advocacy, Canadian Spondylitis Association (CSA), Toronto, Canada; Health & Territory Research (HTR), Universidad de Sevilla, Seville, Spain; Patient Engagement, Novartis Pharma AG, Basel, Switzerland; Health & Territory Research (HTR), Universidad de Sevilla, Seville, Spain; Patient Advocacy, Spanish Federation of Spondyloarthritis Associations (CEADE), Madrid, Spain

**Keywords:** Axial spondyloarthritis, phenotype, regional differences, diagnostic

## Abstract

**Objectives:**

To explore differences in axial spondyloarthritis (axSpA) clinical phenotype around the world in a large sample of patients included in the International Map of Axial Spondyloarthritis (IMAS).

**Method:**

IMAS was a cross-sectional online survey (2017–2022) of 5557 unselected axSpA patients from 27 countries. We analysed across five geographic regions the age at symptom onset, diagnostic delay, gender, HLA-B27, family history, extra-musculoskeletal manifestations, presence of comorbidities, disease activity (BASDAI), level of spinal stiffness and treatments.

**Results:**

Of 5557 IMAS participants, 3493 were from Europe, 770 from North America, 600 from Asia, 548 from Latin America and 146 from South Africa. Age at symptom onset ranged between 25 and 30 years and was higher in Latin America. Diagnostic delay was longest in South Africa and lowest in Asia. The lowest HLA-B27 positivity was observed in Latin America and the highest in Asia. Extra-musculoskeletal manifestations were the lowest in Europe. Mean disease activity (BASDAI) was 5.4, with highest values in South Africa and lowest in Asia. Most of the patients had used NSAIDs for their condition and less than half had ever taken conventional synthetic DMARDS; both were more frequent in Latin America and South Africa. Almost half of the patients had ever taken biologic DMARDs, more frequent use being in the Americas.

**Conclusion:**

There is great heterogeneity of axSpA clinical phenotype presentation around the world. AxSpA manifests differently in different regions, so further understanding of these differences of phenotypes is needed to achieve early diagnosis and initiation of optimal disease treatment in axSpA in the different regions.

Rheumatology key messages

- Patients in South Africa present a higher disease burden probably triggered by their almost 11 years of diagnostic delay.
- North America has the highest prevalence of uveitis and psoriasis, while the highest proportion of patients with IBD and physical comorbidities was found in South Africa.
- HLA-B27 test was positive in less than 80% of patients in all regions.

## Introduction

Phenotype is the observable expression of an individual's genotype in the form of appearance, signs and symptoms [1] and is essential in genetic studies aimed at identifying disease genes [2]. Axial spondyloarthritis (axSpA) is a heterogeneous chronic disease with a predilection for the axial skeleton [3] that may be manifested and frequently designated as radiographic compared with non-radiographic axial spondyloarthritis (nr-axSpA) [4].

According to a systematic review, age at symptom onset of patients with axSpA range between 20 and 35 years, with the most common clinical feature being inflammatory back pain [5]. Family history and HLA-B27 test are the main biomarkers used for the diagnosis of axSpA [6]. In this context, a review study including the ASAS, DESIR and SPACE cohorts in axSpA showed a positive family history in 23–39% of patients, while HLA-B27 test was positive in 43–58% of patients [7]. The combination of a positive HLA-B27 test and severe sacroiliitis in patients with early inflammatory back pain is a possible indicator for the presence of axSpA [8]. In this context, a recent study showed HLA-B27 test positivity in 69.1% of axSpA patients and 56.0% for non-radiographic patients (nr-axSpA). This test showed a higher percentage of positivity in Asian patients (80.3%), followed by Latin Americans (67.9%), Americans (61.2%) and Europeans (57.6%) [9]. Unfortunately, these percentages are still too low for the rheumatologist to achieve an early and effective diagnosis.

Patients with axSpA frequently experience peripheral and extra-musculoskeletal manifestations [10], with around 20% of patients suffering from uveitis [11], between 4 and 12% from inflammatory bowel disease (IBD) [12] and 16.7% from psoriasis [13].

It is important to investigate the phenotype characteristics of patients with axSpA as there are still many unresolved questions relating to the genetic mechanisms that trigger the disease onset. Understanding the characteristics of axSpA patients based on gender, race/ethnicity or region would allow individualizing the diagnostic approach and treatment of patients based on their phenotypic characteristics. Because of the challenge for rheumatologists to diagnose, treat and manage patients with axSpA, increased knowledge about the phenotype could be useful to identify potential axSpA cases earlier through genetic testing. This would help precision medicine to reduce diagnostic delay, prescribe individualized drugs and better predict health outcomes including disease progression.

Most of the published cohort studies are restricted to particular regions mainly in the European and North American continents, which precludes inter-region comparisons and neglects the phenotypic variability around the world.

The present analysis aims, therefore, to explore the axSpA phenotype in different geographical regions in a large sample of patients included in the International Map of Axial Spondyloarthritis (IMAS).

## Methods

### Study design and survey development

The IMAS initiative started in 2017 with the Spanish Atlas of Axial Spondyloarthritis study [14]. Subsequently, 12 European countries joined the project, which became the European Map of Axial Spondyloarthritis (EMAS) [15]. Finally, countries from all over the world joined the project, which became known as IMAS, resulting in a final cohort of 27 countries. The questionnaire was first developed for the 2017 Atlas [14] and translated into the different IMAS countries’ languages.

### Settings

IMAS collected information through an online cross-sectional survey (2017–2022) including unselected axSpA patients from Europe, Asia, North America, Latin America and South Africa. The questionnaire was administered via an online survey platform managed by Ipsos. This questionnaire was translated into the main language of each of the 27 participating IMAS countries. The specific information about the questionnaire can be found in the seminal EMAS article [15].

### Participants and recruitment

The sample eligibility criteria were: age ≥18 years, residence in one of the specified countries, and a self-reported diagnosis of axSpA (either AS or nr-axSpA).

### Variables

In the present study, variables related to phenotype are reported. The measurement and categories of variables are shown in Supplementary Table S1, available at *Rheumatology* online.

Patient-reported outcomes were collected from the following scales:

- Spinal Stiffness Index: an index developed by the University of Seville specifically for the IMAS survey to assess the degree of spinal stiffness. The index has a range from 3 to 12. More detailed information is provided in the seminal article [14, 15].
- Functional Limitation Index: an index developed by the University of Seville specifically for the IMAS survey to assess the degree of limitation in activities of daily life. The index has a range from 0 to 54. More detailed information is provided in the seminal article [14, 15].
- Bath Ankylosing Spondylitis Disease Activity Index (BASDAI): a self-administered questionnaire that evaluates disease activity in patients with axSpA. It includes six questions relating to the following symptoms: fatigue; pain in the spinal column; inflammation/pain in joints other than the neck, back and hips; areas of localized tenderness (also called enthesitis, or inflammation of tendons and ligaments); and the level and duration of stiffness in the morning—all assessed on a 0–10 numeric rating scale [15]. The overall BASDAI has a range from 0 to 10.

### Study size

**Figure 1.**
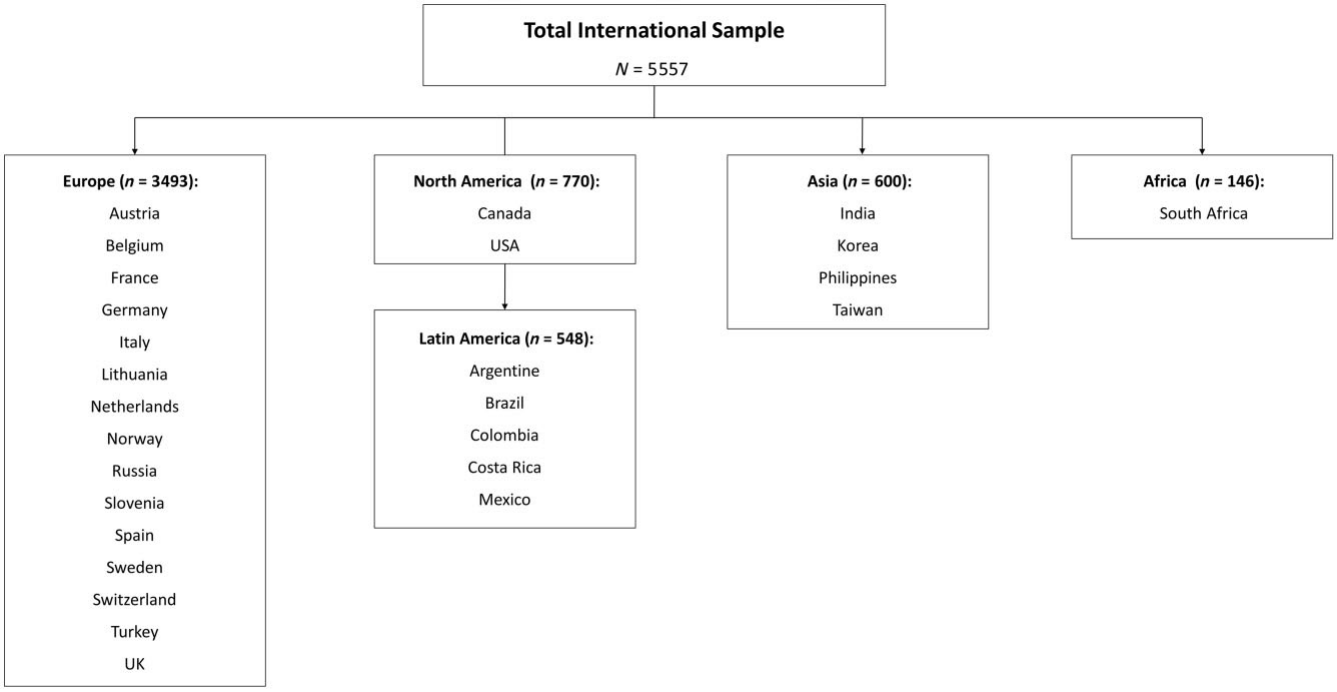
Flowchart showing the participating countries

A total of 5557 patients with axSpA participated in the IMAS. The variables analysed in each region are available in Fig. 1.

### Statistical analysis

For those variables with missing values, reduced sample sizes are reported in order to eliminate unwanted bias. The results are presented as summary statistics, with mean and s.d. for continuous variables, and frequency and percentages for categorical variables. The sample size available for each of the variables (denoted by *n*) is specified, while *N* is used to designate the global sample (*N* = 5557). In the bivariate analysis, Kruskal–Wallis tests were used to evaluate homogeneity in the distribution of regions (Europe, Asia, North America, Latin America and South Africa) and quantitative variables. The χ^2^ test was used to evaluate the differences between regions and categorical variables. All comparisons were considered statistically significant when *P* < 0.05. Data analysis was conducted using SPSS V.26.0 (IBM Corp., Armonk, NY, USA).

### Ethics declarations

The present manuscript does not contain any studies with animal subjects. All participants were asked to provide explicit opt-in consent prior to participating in the IMAS survey. Furthermore, the participants' data were anonymized and did not contain confidential, personal or subject-identifying information. Ethical aspects related to data extracted from patients and their treatment were in accordance with the Declaration of Helsinki.

## Results

The number of axSpA patients participating in IMAS was 5557. The mean age was 43.9 years, 55.4% were females and 46.2% had completed university education. Mean age at symptom onset was 26.8 years, and there was a mean symptom duration of 17.1 years and a mean diagnostic delay of 7.4 years. The proportion of patients who were HLA-B27 positive was 71.1% and 36.1% had family history; 23.2% suffer from uveitis, 14.0% from IBD and 20.4% from psoriasis. Almost 70% had at least one physical comorbidity. The mean disease activity, spinal stiffness and functional limitation were 5.4 (out of 10), 7.5 (out of 12) and 19.8 (out of 54), respectively. With respect to treatments, 78.5% had taken NSAIDs and almost half had taken conventional synthetic DMARDs (csDMARDs) or biologic DMARDs (bDMARDs) (Table 1).

**Table 1.** The clinical phenotype of axial spondyloarthritis stratified by region (*N* = 5557)

**Variables**	*n*	Value
Gender, female, *n* (%)	5555	3080 (55.4)
Age, mean (s.d.), years	5555	43.9 (12.8)
Age at symptom onset, mean (s.d.), years	5457	26.8 (11.3)
Symptom duration, mean (s.d.), years	5421	17.1 (13.3)
Diagnostic delay, mean (s.d.), years	5327	7.4 (9.0)
HLA-B27, positive, *n* (%)	3465	2464 (71.1)
Family history of axSpA, *n* (%)	3351	1211 (36.1)
Uveitis, *n* (%)	5050	1171 (23.2)
IBD, *n* (%)	5156	724 (14.0)
Psoriasis, *n* (%)	2265	461 (20.4)
Physical comorbidities, *n* (%)	5449	3720 (68.3)
BASDAI (0–10), mean (s.d.)	4995	5.4 (2.1)
Spinal stiffness (3–12), mean (s.d.)	5371	7.5 (2.5)
Functional limitation (0–54), mean (s.d.)	5482	19.8 (15.4)
NSAIDs, ever, *n* (%)	4995	3921 (78.5)
csDMARDs, ever, *n* (%)	4922	2137 (43.4)
bDMARDs, ever, *n* (%)	5034	2456 (48.8)

axSpA: axial spondyloarthritis; BASDAI: Bath Ankylosing Spondylitis Disease Activity Index; bDMARDs: biologic DMARDs; csDMARDs: conventional synthetic DMARDs.

**Figure 2.**
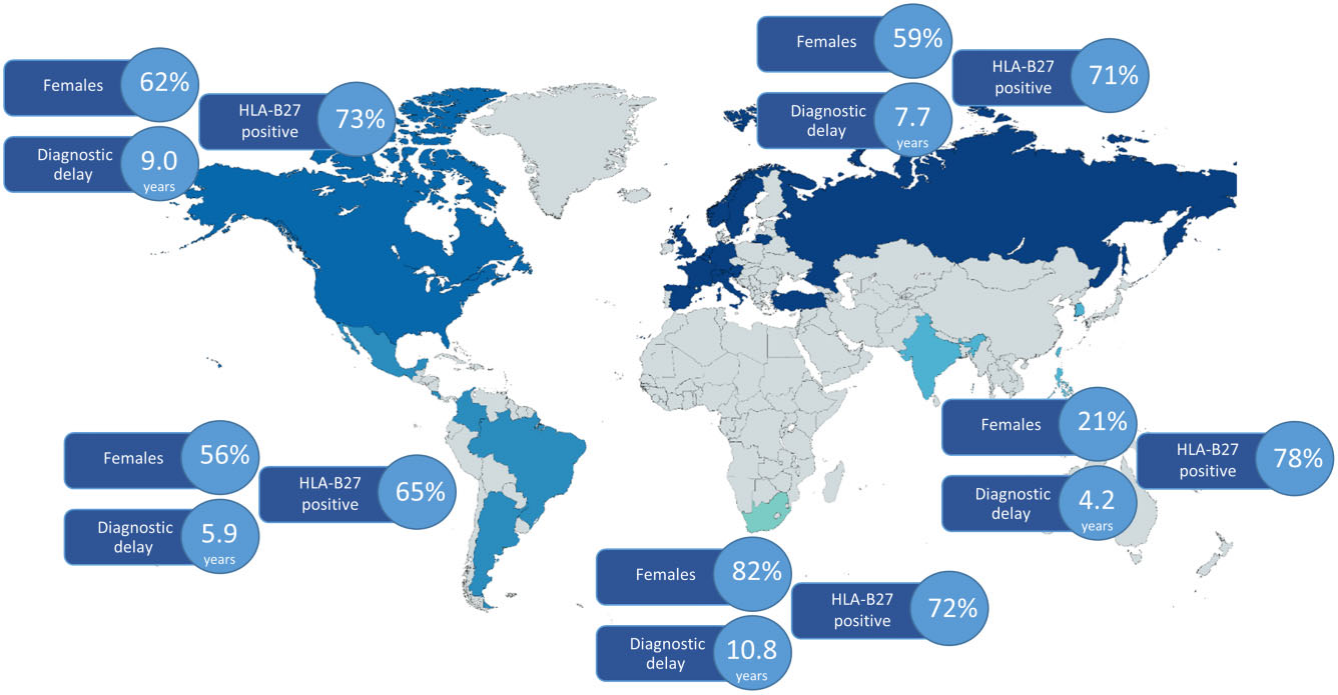
Gender, diagnostic delay and HLA-B27 test in patient with axial spondyloarthritis stratified by region (*N* = 5557)

The proportion of females was highest in South Africa (82.2%), followed by North America, Europe and Latin America (62.3%, 58.7% and 56.0%, respectively), and was much lower in Asia (20.8%; *P* < 0.001). Diagnostic delay was the shortest in Asia and Latin America (4.2 and 5.9 years, respectively), followed by Europe and North America (7.7 and 9.0 years, respectively), and was much longer in South Africa (10.8 years; *P* < 0.001). The HLA-B27 test was positive in a higher proportion in Asia (78.2%) compared with Latin America, Europe, Asia and North America (65.1%, 70.6%, 72.2% and 73.0%, respectively; *P* = 0.005; Fig. 2).

**Figure 3.**
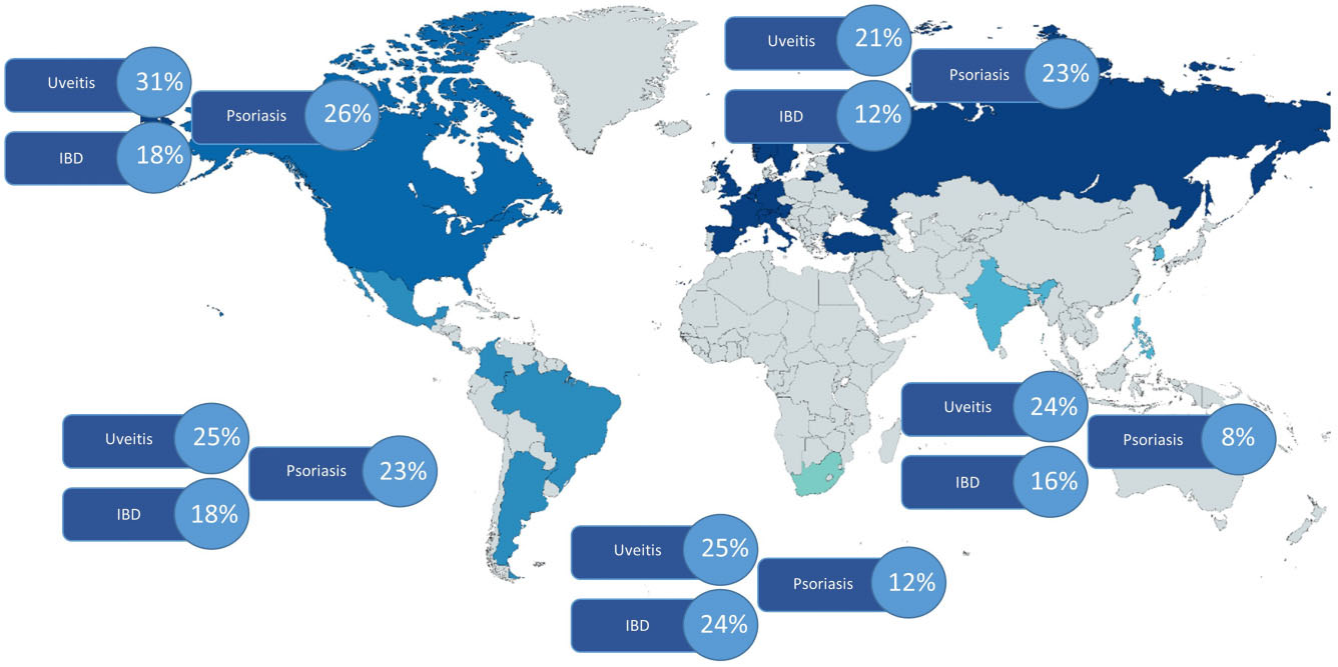
Extra-musculoskeletal manifestations in axial spondyloarthritis stratified by region (*N* = 5557)

With respect to extra-musculoskeletal manifestations, 30.8% of patients in North America had uveitis compared with 21.1% in Europe. Furthermore, in Europe only 11.9% present with IBD compared with a higher ratio in South Africa, North America, Latin America and Asia (24.1%, 18.3%, 17.5% and 16.1%, respectively). Finally, only 11.9% of patients suffer from psoriasis in South Africa, the percentage being much higher in North America (26.3%), Europe (23.0%) and Latin America (22.9%) (Fig. 3).

Age at symptom onset was higher in Latin America (30.5 years) than in the rest of the regions (26 years). Patients in Europe, North America and South Africa had been living with the condition for an average of 18 years, while this figure was lower in Asia (11.6 years) and Latin America (13.7 years). Nearly 40% of patients in Europe have family history, followed by North America (32.6%), Latin America (28.7%) and Asia (22.3%). Between 80% and 90% of patients in Latin America and South Africa had at least one physical comorbidity, followed by 73.8% in North America and only 62.7% and 64.6% in Asia and Europe, respectively. Disease activity (BASDAI) was the highest in South Africa (6.0), followed by Latin America and North America (5.6 and 5.5, respectively) and Europe (5.4), and was lowest in Asia (4.9). Spinal stiffness was highest in South Africa (8.6), followed by Europe and North America (7.8 and 7.6, respectively), and lowest in Asia and Latin America (6.9 and 6.6, respectively). In North America patients reported the highest functional limitations (23.0), followed by Europe, South Africa and Latin America (20.3, 19.1 and 18.9, respectively), with the lowest functional limitation in Asia (13.8) (all *P*-values <0.001). With respect to the use of NSAIDs, >90% had taken them in South Africa and Latin America, followed by North America (82.6%) and about 75% in Europe and Asia. The use of csDMARDs was most frequent in South Africa (79.3%), followed by Latin America (65.4%) and Asia (52.3%), while Europe and North America had the lowest proportions (36.9% and 40.2%, respectively). Finally, the use of bDMARDs was most frequent in Latin America and North America (65.5% and 61.8%, respectively), followed by Europe (45.0%) and South Africa (42.5%), with the lowest proportion in Asia (37.0%) (all *P*-values <0.001; Table 2).

**Table 2.** Diagnostic characteristics, extra-musculoskeletal manifestations, comorbidities, patient-reported outcomes and treatments of patients with axial spondyloarthritis stratified by region (*N* = 5557)

Characteristic	Europe	North America	Latin America	Asia	South Africa	*P*-value
	(*n* = 3493, 62.9%)	(*n* = 770, 13.9%)	(*n* = 548, 9.9%)	(*n* = 600, 10.8%)	(*n* = 146, 2.6%)	
Age at symptom onset, mean (s.d.), years	26.2 (10.8)	26.4 (12.1)	30.5 (12.9)	26.9 (10.7	26.7 (11.4	<0.001
Symptom duration, mean (s.d.), years	18.4 (13.6)	18.0 (14.4)	13.7 (11.2)	11.6 (9.3	18.0 (12.7	<0.001
Family history of axSpA^a^, yes, *n* (%)	937 (39.0)	138 (32.6)	86 (28.7)	50 (22.3)	—	<0.001
Physical comorbidities, yes, *n* (%)	2186 (64.6)	568 (73.8)	458 (83.6)	376 (62.7)	132 (90.4)	<0.001
BASDAI (0–10), mean (s.d.)	5.4 (2.1)	5.5 (2.2)	5.6 (2.5)	4.9 (2.0)	6.0 (1.8)	<0.001
Spinal Stiffness (3–12), mean (s.d.)	7.8 (2.5)	7.6 (2.3)	6.6 (2.6)	6.9 (2.5	8.6 (1.8	<0.001
Functional limitation (0–54), mean (s.d.)	20.3 (15.9)	23.0 (14.7)	18.9 (14.9)	13.8 (12.3)	19.1 (13.1)	<0.001
Use of NSAIDs, ever, *n* (%)	2224 (75.4)	633 (82.6)	490 (90.1)	438 (74.7)	136 (94.4)	<0.001
Use of csDMARDs, ever, *n* (%)	1069 (36.9)	305 (40.2)	356 (65.4)	298 (52.3)	111 (79.3)	<0.001
Use of bDMARDs, ever, *n* (%)	1337 (45.0)	476 (61.8)	359 (65.5)	222 (37.0)	62 (42.5)	<0.001

a Family history: this information was not collected in South Africa. axSpA: axial spondyloarthritis; BASDAI: Bath Ankylosing Spondylitis Disease Activity Index; bDMARDs: biologic DMARDs; csDMARDs: conventional synthetic DMARDs.

**Figure 4.**
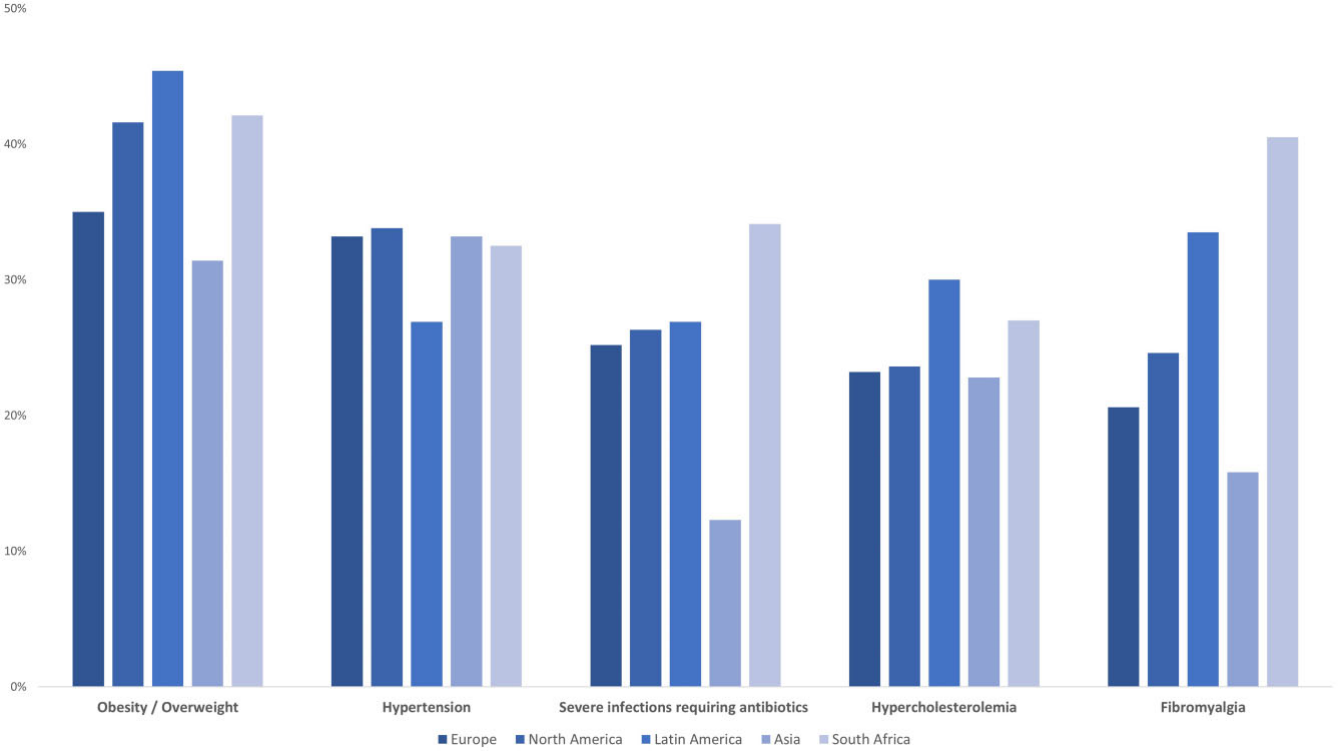
The most frequent comorbidities of patients with axial spondyloarthritis stratified by region (*N* = 5557)

Figure 4 shows the most frequent comorbidities stratified by region, with the two most frequent comorbidities being obesity/overweight and hypertension in Europe (35.0% and 33.2%, respectively), North America (41.6% and 33.8%, respectively) and Asia (31.4% and 33.2%, respectively), and obesity/overweight and fibromyalgia in Latin America (45.4% and 33.5%, respectively) and South Africa (42.1% and 40.5%, respectively) (Fig. 4).

## Discussion

The IMAS study provides evidence on the phenotypic characteristics of patients with axial axSpA in different regions globally. Our study has shown differences in the phenotype of 5557 axSpA patients from Europe, Asia, North America, Latin America and South Africa. These data show an unacceptable diagnostic delay, presence of extra-musculoskeletal manifestations and poor patient-reported outcomes in a large cohort of axSpA patients worldwide.

In particular, the results obtained allow the phenotypic profile of patients with axSpA in each of the regions to be established. Specifically, in Europe, longer symptom duration and lower use of csDMARDs were observed. In North America patients had high diagnostic delay and symptom duration, as well as a high presence of uveitis, psoriasis and functional limitations. In Latin America, axSpA patients had the highest age at symptom onset and the lowest HLA-B27 positive, physical comorbidities and spinal stiffness. In Asia there was the shortest diagnostic delay, as well as the lowest proportion of females, physical comorbidities, symptom duration and family history, but also the highest HLA-B27 positivity. In addition, Asian patients had the lowest disease activity, functional limitations, and the lowest use of NSAIDs and bDMARDS. South Africa had the highest proportion of females, diagnostic delay, IBD, physical comorbidities, disease activity, spinal stiffness, and use of NSAIDs and csDMARDS, but the lowest proportion of psoriasis.

This knowledge about differences in the phenotype of axSpA patients across regions generated in the present study is essential for clinical practice, especially for early diagnosis as well as optimal treatment and management.

The first differences between regions are found in the proportion of female patients, their being the majority in South Africa (82%) and the lowest in Asia (21%). Previously, axSpA was considered to be a predominantly male disease [16], but more recent evidence has shown it is also prevalent among females [17], possibly due to the improvement in diagnosis of non-radiographic disease where females are more prevalent [18]. It is worth noting the high prevalence of females in IMAS in South Africa along with the longest diagnostic delay of all the regions analysed (10.8 years). At the same time, the South African patient population was the smallest in the IMAS sample, which is associated with the highest risk of bias. Moreover, this longer diagnostic delay in South African patients—mostly females—could be the cause of the worse patient-reported outcomes shown as well as the appearance of physical and extra-musculoskeletal manifestations.

With respect to diagnostic characteristics, the oldest age at symptom onset occurred in Latin America (30 years of age), while in the other regions it was around 26 years. A meta-analysis showed that the age at symptom onset ranged from 20 and 35 years [19], which is in line with the IMAS cohort. Therefore, non-rheumatology medical specialists should consider the presence of back pain in young adults as a possible indicator of axSpA.

The mean symptom duration among IMAS patients was 17 years, although it was only 11.6 years in Asia and 13.7 years in Latin America. A multi-regional study in axSpA patients found the shortest symptom duration in China and in Latin America, although it was much longer in Canada [9]. The overall mean diagnostic delay for IMAS patients was about 7.4 years, the highest being in South Africa and the lowest in Asia. This is slightly longer than assessed by a meta-analysis of 64 studies that reported a mean diagnostic delay of 6.7 years and the systematic review of 69 studies by Hay reporting a median delay of between 2 and 6 years [19, 20]. The diagnostic delay of patients with axSpA is unacceptable and may affect the progression of the disease and consequently the burden on the patients. This delay may be due to lack of awareness of axSpA by primary care physicians, and failure to refer these patients to a rheumatologist—the medical specialist specially trained in diagnosing axSpA. In addition, access to these medical specialists may differ by region, as not all countries in each of the IMAS regions have the same healthcare system with the same possibilities for access to medical care and test performance, and not all patients can economically afford to use a private healthcare system. Therefore, all this may explain the heterogeneity of diagnostic delay across the regions evaluated in IMAS.

The HLA-B27 test was positive in >70% of IMAS patients, the lowest being in Latin America (65%) and the highest in Asia (78%). Although the proportion of HLA-B27-positive patients in Europe and North America was lower than reported in a previous study by Poddubnyy *et al.*, the results from Asia (80%) and Latin America (68%) were similar [9]. One-third of the patients had another family member with the disease, with the highest proportion in Europe and the lowest in Asia. However, this figure was below 20% in the study by Poddubnyy *et al.* [9]. Therefore, knowledge of the patients' phenotype in their region, together with more effective disease biomarkers, could reduce the diagnostic delay for axSpA patients.

Of note, the majority of patients in Asia are male, with the highest proportion of HLA-B27 positive and with the shortest diagnostic delay of all regions analysed (4.2 years). This could be due to the confidence of specialists in Asia about the utility of the HLA-B27 test to diagnose axSpA combined with the assumption bias that this disease is predominantly a male disease. At the same time this might be an indicator that axSpA patients who are HLA-B27 negative, female, or have milder disease might be underdiagnosed. Regarding extra-musculoskeletal manifestations in IMAS participants, 23% had uveitis, 20% psoriasis and 14% IBD. The highest prevalence of uveitis and psoriasis was in North America (31% and 26%, respectively), while for IBD the highest prevalence was in South Africa (24%). Similarly, the study by Poddubnyy *et al.* conducted in different regional groups showed that the percentage of uveitis in Canada and Latin America was close to 20% [9], and patients in Canada presented with the highest prevalence of psoriasis at 14%. However, the prevalence of IBD in patients with axSpA evaluated in a meta-analysis by Stolwik *et al*. and a review by van de Horst Bruinsma and Nurmohamed is found to be below 10% [21, 22]. Therefore, our results show that IBD manifestations are more frequent in patients with axSpA than previous studies have shown, and since IBD manifestations are associated with increased disease activity, axSpA should also be considered [23].

Sixty-eight percent of IMAS patients had physical comorbidities. South Africa and Latin America had a higher proportion of patients with physical comorbidities. A recent meta-analysis also showed that the other most prevalent comorbidities in patients with axSpA were hypertension, hyperlipidaemia and obesity [24]. These results show that axSpA patients, in addition to having to live with this chronic disease, must cope with several comorbidities and extra-musculoskeletal manifestations.

For patient-reported outcomes, axSpA patients in South Africa had higher disease activity and spinal stiffness, whereas disease activity in Asia and spinal stiffness in Latin America were the lowest. Asian patients presented the least functional limitation, while North American patients were the most functionally limited. These results are consistent with the study by Poddubnyy *et al.* showing that axSpA patients in China had the lowest disease activity compared with other regions of the world [9].

With respect to treatments, patients in South Africa had used NSAIDs and csDMARDs more frequently, while bDMARDs were used more frequently in the Americas. It is noteworthy that in Asia we found the lowest proportion of use of NSAIDs and bDMARDs. This heterogeneity in the use of different drugs to treat axSpA in the different IMAS regions could be due to limitations in access to these drugs in the countries or the lack of effectiveness of these drugs, leading to the use of other drugs.

However, the IMAS study is not without limitations. First, the study was based on self-reported data, and we were unable to confirm participants' diagnoses or corroborate their responses with clinical evaluation. In addition, no classification criteria were used to recruit patients in the study. Moreover, the diagnostic criteria used in each of the regions may differ. In addition, the use of non-validated scales or indices to assess functional limitations in activities of daily living and spinal stiffness should be considered when assessing the results. Comorbidities, specifically extra-musculoskeletal manifestations, were self-reported by patients, so the prevalence may be slightly higher than expected. The ethnicity for the IMAS subjects was predominantly white or Asian and therefore we cannot apply these results to the black population or people from the Middle East. Thus, this should be taken into account when generalizing the results obtained. Despite these limitations, this global analysis on phenotype of axSpA will enable more detailed investigations to obtain evidence on the critical issues that matter to patients, including regional differences.

## Conclusions

The results of this large cohort of people with axSpA show great heterogeneity in the presentation of the clinical phenotype of axSpA across regions. Patients in South Africa present a higher disease burden probably triggered by their almost 11 years of diagnostic delay, while Asian patients – with only 4 years of diagnostic delay – have a lower disease burden. North America has the highest prevalence of uveitis and psoriasis, while the highest proportion of patients with IBD and physical comorbidities was found in South Africa. In contrast, Europe had the lowest proportion of patients with uveitis and IBD. The HLA-B27 test was positive in <75% of patients in all regions, which is too low a rate for a diagnostic test aimed at identifying patients with axSpA. Knowledge of the phenotypic characteristics of patients in different regions is vital for early diagnosis, early initiation of disease treatment and improved management of axSpA patients. Therefore, this information may be useful for rheumatologists to reduce the disease burden of their patients.

## Supplementary Material

kead665_Supplementary_Data

## Data Availability

Contact the corresponding author for availability of data.
