# Supplementary material for: Regional differences in clinical phenotype of axial spondyloarthritis: results from the International Map of Axial Spondyloarthritis (IMAS)
Source: Rheumatology (Oxford). 2023 Dec 21;63(9):2328–35. doi: 10.1093/rheumatology/kead665 (PMC11371368; doi:10.1093/rheumatology/kead665)
Supplement: kead665_Supplementary_Data [file kead665_supplementary_data.docx]

**Supplementary material**

**Supplementary Table S1.** Variables, questions and categories/measurements included in this analysis.

| **Variables** | **Questions** | **Categories/measures** |
| --- | --- | --- |
| **Socio-demographic** | | |
| Gender | Please specify your gender | Male, female |
| **Diagnostic characteristics** | | |
| Age at symptom onset | Age of onset of first symptoms (pain, inflammation, stiffness) atlas associated with Spondylitis/ Spondyloarthritis | In years |
| Diagnostic delay | Calculated based on the age at diagnosis | In years |
| Symptom duration |  | In years |
| HLA-B27 | What was the result of the genetic test (HLA-B27)? | Positive, negative |
| Family history of axSpA | Do you have anyone else in your family suffering from Spondylitis / Spondyloarthritis? | Yes, no |
| **Disease extra-musculoskeletal manifestations** | | |
| Uveitis | Please indicate whether you have been diagnosed with any of the following: | Uveitis |
| Inflammatory bowel disease |  | Inflammatory bowel disease |
| Psoriasis |  | Psoriasis |
| **Medication** | | |
| NSAIDs | Have you ever been treated with a Non-Steroidal Anti-Inflammatory Drug (NSAID) for your Spondylitis / Spondyloarthritis? | Yes, no |
| bDMARDs | Have you ever been treated with a Biologic for your Spondylitis / Spondyloarthritis? | Yes, no |
| csDMARDs | Have you ever been treated with a Disease Modifying Anti-rheumatic Drug (DMARD) for your Spondylitis / Spondyloarthritis? | Yes, no |
| **Physical comorbidities** | | |
| Physical comorbidities | Please indicate whether you have been diagnosed with any of the following: | List of 22 physical comorbidities |

NSAIDs: Non-steroidal anti-inflammatory drugs; bDMARDs: Biological disease-modifying antirheumatic drugs; csDMARDs: Conventional synthetic disease-modifying antirheumatic drugs. Physical comorbidities including: atherosclerosis, cataracts, coronary artery disease, diabetes, fibromyalgia, genital lesions, glaucoma, gout, heart failure, hypercholesterolemia, hypertension, irregular heartbeat, karzinome, kidney failure, liver disease, obesity or overweight, osteoporosis, pacemaker fitted, psoriasis, severe infections requiring antibiotics, severe infections requiring hospital admission, and spinal or other fractures.
